# Intravascular Ultrasound Guiding Percutaneous Coronary Interventions in Complex Higher Risk-Indicated Patients (CHIPs): Insight from Clinical Evidence

**DOI:** 10.31083/j.rcm2512443

**Published:** 2024-12-18

**Authors:** Sidonio Mesquita Viana, Dai-Min Zhang

**Affiliations:** ^1^Department of Cardiology, Sir Run Run Hospital, Nanjing Medical University, 211112 Nanjing, Jiangsu, China

**Keywords:** intravascular ultrasound, percutaneous coronary intervention, coronary artery disease, complex higher risk-indicated patients, major adverse cardiovascular events

## Abstract

Intravascular ultrasound (IVUS) in percutaneous coronary intervention (PCI) has transformed the management of complex higher risk-indicated patients (CHIPs), representing a pivotal advancement in high-risk procedure navigation. IVUS, complementing conventional angiography, provides unparalleled insights into lesion characteristics, plaque morphology, and vessel structure, enhancing the precision of stent placement and postprocedural care for CHIPs. The ongoing trials underscore the pivotal role of IVUS in optimizing procedural accuracy and improving clinical outcomes for high-risk patients, promising exciting new findings. However, notable gaps persist, encompassing the absence of standardized IVUS protocols, cost implications, and limited integration into routine practice. This study aims to address these gaps comprehensively by further delineating the influence of IVUS on patient outcomes, procedural success, and long-term prognostic indicators. This review aims to provide a clear overview of IVUS-guided PCI in CHIP, highlighting the significance of ongoing trials, identifying prevalent challenges, and outlining the objective of narrowing these gaps.

## 1. Introduction

Coronary artery disease (CAD) poses a significant health challenge, especially for patients classified as complex higher risk-indicated patients (CHIPs) [1]. These individuals present intricate coronary lesions, multiple comorbidities, and heightened risk, making their management a clinical conundrum. Percutaneous coronary intervention (PCI) has revolutionized the treatment landscape for CAD, offering a minimally invasive approach for revascularization and symptom relief. However, the complexities in the context of CHIP demand a more nuanced and precise intervention strategy. This is where our study, focusing on using intravascular ultrasound (IVUS), a complementary imaging modality to conventional angiography, comes in. Our research provides high-resolution intravascular images, allowing for a comprehensive assessment of lesion morphology, plaque burden, and vessel architecture. This technology offers real-time, detailed visualization of the coronary vasculature, enabling clinicians to discern subtleties that may elude conventional imaging techniques [2].

The essence of IVUS-guided PCI lies in its ability to offer precise insights into lesion characteristics, thus revolutionizing the optimization of stent placement [3]. Unlike angiography, which provides a two-dimensional depiction of the vessel lumen, IVUS enables a three-dimensional assessment to accurately determine vessel size, plaque burden, and morphology. This comprehensive evaluation significantly influences decision-making during PCI procedures, allowing for tailored interventions that match each patient’s needs, particularly in the challenging landscape of CHIPs. The adoption of IVUS in PCI procedures augments conventional angiography-driven interventions by providing clinicians with vital information to navigate complex lesions more effectively [4]. These findings address the limitations inherent in angiography, such as difficulties in assessing vessel remodeling, plaque composition, and stent apposition, which are crucial factors in determining the success and durability of coronary interventions, especially in high-risk patients.

Furthermore, IVUS guidance enhances the precision of stent deployment, optimizing stent sizing, expansion, and apposition within the coronary artery. This meticulous approach reduces the risks associated with suboptimal stent placement, such as stent malapposition, edge dissections, and incomplete lesion coverage, all of which can predispose patients, especially those in the CHIP category, to adverse events and potential procedural failures [5].

In essence, IVUS-guided PCI represents a significant advancement in interventional cardiology, particularly in managing the intricate complexities of CHIP patients [6]. IVUS technology enhances the precision, safety, and efficacy of PCI procedures, ultimately aiming to improve patient outcomes and redefine the standards of care in the challenging landscape of coronary interventions for high-risk individuals.

## 2. Defining CHIP Patients and Unraveling the Complexity

CHIPs represent a distinct subgroup within the spectrum of individuals with CAD. This section aims to delineate the multifaceted criteria defining CHIP, elucidating the heightened complexities, prevalent comorbidities, and elevated risks encountered during coronary interventions [7]. The classification of patients in the CHIP category encompasses a convergence of intricate factors, marking them as a high-risk cohort necessitating specialized attention and tailored therapeutic approaches [8]. The criteria defining CHIPs encompass a range of elements, as described below.

### 2.1 Complex Lesions and Disease Severity

CHIP patients often present with multivessel disease, where CAD affects multiple major coronary arteries or branches. Diffuse, complex lesions spanning multiple vessels signify a more significant disease burden and pose challenges in revascularization strategies [9]. The involvement of the left main coronary artery signifies an advanced and high-risk subset of CAD patients. CHIP patients with left main disease often require intricate interventions due to the critical nature of this vessel. The presence of chronic total occlusions signifies complete blockage in coronary arteries, posing challenges in revascularization due to the technical complexities and increased procedural risks. CHIP patients frequently harbor bifurcation lesions or lesions in anatomically challenging areas, requiring sophisticated interventional techniques and specialized equipment.

### 2.2 Comorbidities and Clinical Complexity

The coexistence of diabetes mellitus significantly elevates the risk profile of CAD patients. CHIP patients with diabetes exhibit heightened complexities, including accelerated atherosclerosis, microvascular disease, and increased susceptibility to adverse outcomes of postintervention [10]. Renal impairment, particularly in the form of chronic kidney disease (CKD), is prevalent in CHIP patients, amplifying the intricacies of their clinical management. Renal dysfunction independently predicts adverse cardiovascular events and complicates treatment strategies [11]. Patients with a reduced ejection fraction, indicative of compromised cardiac function, fall within the purview of CHIP due to their elevated risk and complex coronary anatomy.

### 2.3 Prior History and High-Risk Features

Patients with a history of prior interventions, whether PCI or coronary artery bypass grafting (CABG), constitute a subset at higher risk of restenosis, graft failure, or recurrent disease progression [12]. A history of stent failure or in-stent restenosis places individuals in the CHIP category owing to the increased complexities associated with managing recurrent lesions or complications arising from prior interventions.

## 3. Principles of IVUS Technology

IVUS operates based on high-frequency sound waves and utilizes catheter-mounted ultrasound transducers to generate detailed cross-sectional images of the coronary arteries [13]. The catheter is threaded through the coronary vessels, emitting ultrasound waves and capturing the echoes reflected from the tissues. These echoes are then translated into real-time, high-resolution images, providing a three-dimensional view of the vessel lumen, wall, and surrounding structures. The distinguishing feature of IVUS lies in its ability to offer insights beyond the luminal view provided by conventional angiography. While angiography provides a roadmap of the vessel, it often falls short in characterizing lesion morphology, assessing plaque composition, and evaluating vessel size accurately. On the other hand, IVUS excels in providing detailed information about vessel walls and the nature of atherosclerotic plaques [14].

## 4. Complexities and Risks in Coronary Interventions for CHIP Patients

CHIP patients often present with lesions that are calcified, heavily diseased, or with intricate morphologies. These complexities heighten the technical challenges during interventions, impacting procedural success rates [15]. The intricacies of the lesions and the patient’s underlying conditions predispose them to a higher incidence of procedural complications, such as dissections, perforations, or incomplete revascularization. CHIP patients face increased risks of adverse cardiovascular events postintervention, including stent thrombosis, restenosis, and major adverse cardiac events (MACE). Due to the complexities of their disease and interventions, CHIP patients may exhibit reduced long-term patency rates of stents or grafts, necessitating vigilant follow-up and potential reinterventions [16].

## 5. Applications of IVUS in CHIP-PCI: Navigating the Complexity with Precision

**Fig. 1.**
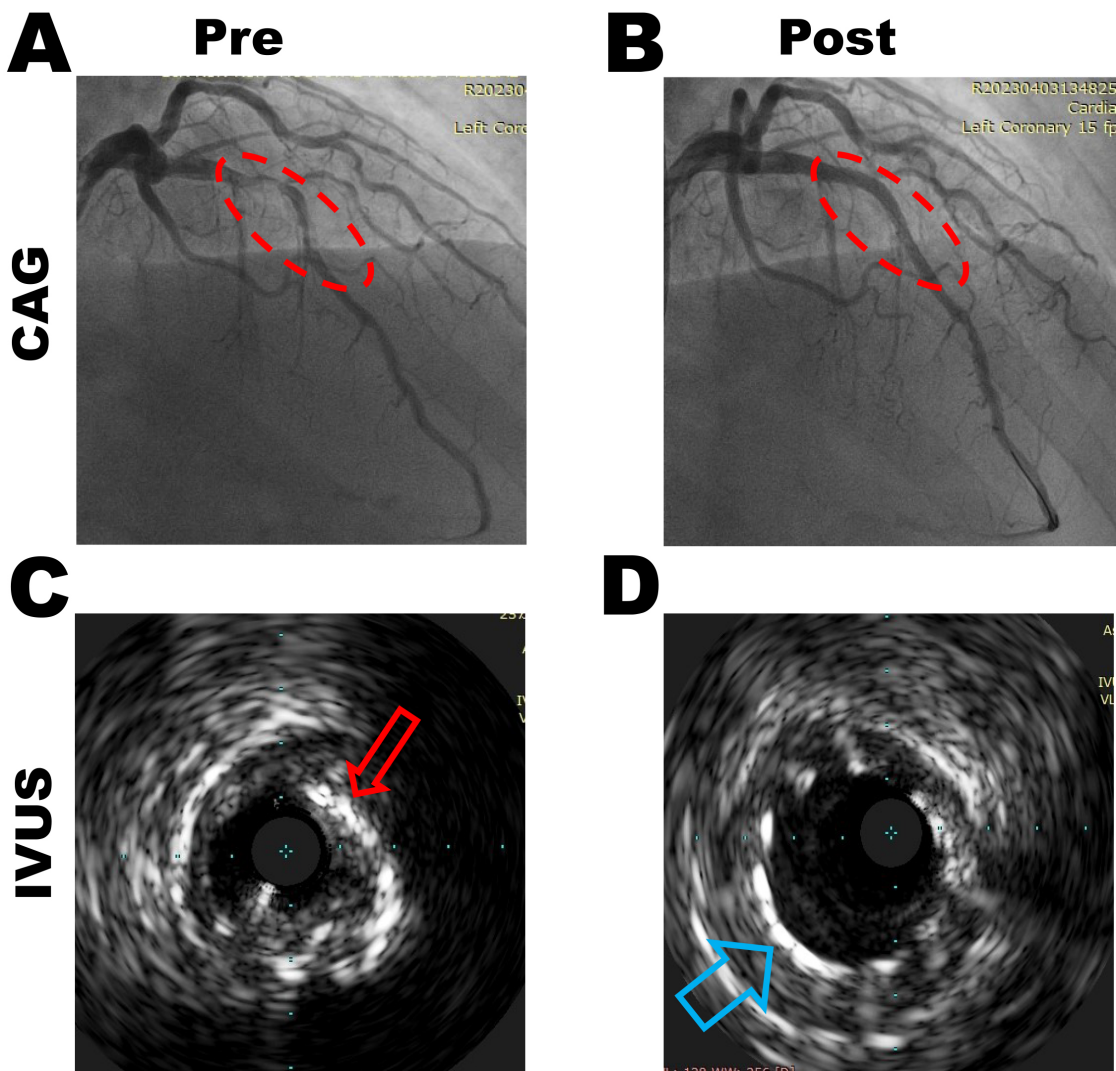
**Intravascular ultrasound guiding percutaneous coronary interventions in a patient with calcified lesion**. (A) Coronary angiography (CAG) image indicated the patients had left anterior descending (LAD) coronary artery diffuse and tortuosity lesions. (C) An intravascular ultrasound (IVUS) image indicated calcified lesions pre-percutaneous coronary intervention (PCI). (B,D) indicated revascularization of LAD post-PCI by CAG and IVUS, respectively. IVUS, intravascular ultrasound; PCI, percutaneous coronary interventions. The red arrow indicates calcified plaques. The blue arrow shows the stent strut post-dilation.

IVUS technology has become a cornerstone in interventional cardiology, particularly PCI [17]. This section delves into the intricate principles and multifaceted applications of IVUS in PCI procedures, emphasizing its pivotal role in assessing lesion morphology, optimizing stent selection, and enhancing procedural success rates, with a specific focus on CHIP. Tailoring interventions to CHIP patients presents a unique set of challenges in PCI procedures, often characterized by complex lesions, multivessel disease, and a greater likelihood of adverse events. With its ability to provide detailed insights into lesion morphology, IVUS is a valuable tool for tailoring interventions to the specific needs of CHIP patients [18]. CHIP patients often present with complex bifurcation lesions. IVUS assists in evaluating the geometry of bifurcation lesions, guiding the selection of appropriate stenting techniques, such as provisional or two-stent strategies. This tailored approach enhances the chances of procedural success in challenging anatomies (see Fig. 1).

### 5.1 Assessing Lesion Morphology

IVUS allows for meticulously examining plaque composition, distinguishing between soft, fibrous, and calcified plaques [19]. This information is crucial in guiding the selection of appropriate interventional strategies, especially for CHIP patients, who often present with complex, heterogeneous lesions. IVUS enables the assessment of vessel remodeling, a critical factor in determining the extent of atherosclerotic disease. The ability to discern positive or negative remodeling aids in planning interventions tailored to the specific characteristics of the lesion [20]. IVUS aids in identifying vulnerable plaques characterized by thin fibrous caps and large lipid cores. These features are associated with an increased risk of plaque rupture and adverse events [21]. Recognizing and addressing these high-risk lesions is crucial in the management of CHIP patients.

### 5.2 Optimizing Stent Selection

IVUS provides precise vessel diameter and lesion length measurements, facilitating accurate stent sizing. This is particularly important in CHIP patients, where vessel size and lesion morphology variations are common. Proper stent sizing ensures adequate lesion coverage and minimizes the risk of stent malapposition or geographic misses. IVUS assists in evaluating the apposition of the stent to the vessel wall, reducing the likelihood of malapposition, which can lead to stent thrombosis or restenosis. This is crucial in CHIP patients, where suboptimal stent deployment can have more profound consequences. IVUS facilitates the selection of an appropriate drug-eluting stent (DES) for CHIP patients based on lesion characteristics [22]. This personalized approach considers factors such as plaque burden, vessel size, and calcifications, ensuring the optimal choice of stent to minimize restenosis and improving long-term outcomes.

### 5.3 Increased PCI Procedural Success in CHIP Patients

By providing real-time feedback on lesion characteristics and the effectiveness of stent deployment, IVUS contributes to reducing procedural complications. This is particularly relevant in CHIP patients, where the risk is heightened due to the complexity of the lesions and the presence of multiple comorbidities. IVUS guidance has been associated with improved long-term outcomes, including reduced MACE. This approach is critical in the high-risk population of CHIP patients, where optimizing procedural success rates directly impacts patient prognosis [23]. Post-PCI assessment for stent expansion: IVUS plays a crucial role in assessing post-PCI expansion. In CHIP patients, where suboptimal stent expansion can lead to adverse events, IVUS provides real-time feedback on the adequacy of stent deployment, allowing immediate adjustments if necessary [24].

In conclusion, IVUS technology has redefined the landscape of PCI procedures, particularly in addressing the complexities of CHIP patients. Its ability to offer detailed insights into lesion morphology, optimize stent selection, and enhance procedural success rates positions IVUS as an indispensable tool in the interventional armamentarium [25]. As we delve deeper into the era of precision medicine, IVUS-guided PCI stands as a beacon of personalized care, navigating the intricate landscape of CHIP patients with meticulous accuracy and improved clinical outcomes.

## 6. Advantages of IVUS Guidance in PCI for CHIP Patients

IVUS provides real-time, high-resolution imaging, allowing precise assessment of lesion morphology, guiding optimal stent sizing, and ensuring accurate stent deployment. This approach results in better lesion coverage and reduced geographic misses, which enhances procedural accuracy, especially for complex lesions characteristic of CHIP patients [26]. The meticulous evaluation of vessel dimensions and plaque characteristics by IVUS minimizes complications such as stent malapposition, edge dissections, and incomplete stent apposition [27]. This decreases the likelihood of adverse events during and after the procedure, fostering better safety profiles in high-risk patients.

IVUS-guided PCI correlates with improved long-term outcomes by reducing the incidence of restenosis, target lesion revascularization, and MACE. Optimized stent placement and reduced complication rates improve patient prognosis and quality of life. Integrating IVUS guidance into PCI procedures for CHIP has become a focal point in interventional cardiology.

### 6.1 Procedural Success Rates: Insights from Clinical Trials

The RENOVATE-COMPLEX PCI trial [28] is a large multicenter trial conducted in South Korea that enrolled patients with complex coronary lesions who were randomized at a 2:1 ratio to undergo either IVUS-guided PCI or angiography-guided PCI. The choice between IVUS and optical coherence tomography (OCT) was at the operator’s discretion. The primary endpoint was a composite endpoint of target vessel failure (TVF), which included cardiovascular death, target-vessel MI, or clinically driven TVR. A total of 1600 patients underwent randomization. Compared with angiography alone, IVUS-guided PCI demonstrated a 36% reduction in the incidence of TVF. There were no differences in procedural safety events between the groups. This finding reiterates prior knowledge that IVUS enhances clinical outcomes among patients with complex coronary lesions undergoing PCI [28]. However, the trial was unblinded, with only 45.4% of patients experiencing intravascular imaging-defined stent optimization. That study also revealed that the proportion of target lesions evaluated by intravascular imaging before intervention was low, and the trial included patients from a single center.

The IVUS-XPL trial [29] was the first large-scale, randomized trial to evaluate the efficacy of IVUS-guided PCI compared to angiography-guided PCI in patients receiving newer generation DESs for complex coronary lesions (defined as implanted stents ≥28 mm in length). The trial enrolled 1400 patients and demonstrated that IVUS-guided stent implantation significantly reduced major adverse cardiac events up to 5 years compared to angiography-guided stent implantation. Sustained 5-year clinical benefits were observed within one year and from 1 to 5 years post-implantation [29]. That study had limitations due to its randomized design, use of a single type of DES, inclusion of only long lesions, low event rate, and 15% loss to follow-up.

The OCTIVUS trial [30] was the most recent randomized trial comparing the impact of IVUS and OCT on complex clinical outcomes in 2000 patients with significant coronary lesions. OCTIVUS achieved its principal goal of lowering significant procedural difficulties while maintaining the same level of imaging-related complications. Notably, 60% of the patients had diffuse long lesions, 11% had left main coronary disease, and 55% had bifurcation lesions. Compared to OCT, IVUS more frequently satisfied the predefined imaging-guided stent optimization parameters. The primary endpoint and its constituent parts were less common in OCT images. The fact that the overall incidence rate was lower than anticipated demonstrated how interchangeable various imaging modalities are [30].

While these trials offer promising insights, ongoing trials such as the IVUS-CHIP, OPTIMUM-IVUS, and OPTIMAL-CTO trials (These clinical trial information be provided at https://clinicaltrials.gov/) are poised to provide more nuanced data on the impact of IVUS-guided PCI in challenging subsets, potentially shedding light on its benefits in CHIP patients.

Previous studies indicated that IVUS improves clinical outcomes in patients with chronic coronary syndrome. However, insufficient data are available concerning the advantages of IVUS guidance for patients with acute coronary syndromes (ACS). Li X *et al*. [31] investigated whether IVUS guidance improves PCI outcomes in patients presenting with ACS compared with coronary angiography (CAG) guidance. In this study, 3505 patients with ACS were randomly assigned to IVUS-guided PCI or CAG-guided PCI. The primary endpoint occurred in 70 patients of the IVUS group and 128 patients of the CAG group driven by reductions in target vessel MI or TVR. These results indicated that in patients with ACS, IVUS-guided PCI resulted in a lower 1-year rate of the composite outcome of cardiac death, target vessel myocardial infarction, or clinically driven revascularisation compared with CAG guidance alone [31]. In CTO-PCI with DES, Kwon *et al*. [32] evaluated the clinical benefits of performing post-stent IVUS in preventing adverse clinical events. They included 1077 patients with 1077 coronary chronic total occlusion (CTO) lesions treated with DES. Post-stent IVUS evaluation was associated with a lower risk of target lesion revascularization (TLR). Taken together, IVUS-guided PCI decreased adverse clinical events.

A meta-analysis [5] conducted ten randomized controlled trials comprising 3452 patients in the IVUS group and 2916 patients in the angiography group who underwent complex PCI. The mean follow-up duration was two years. Compared with angiography PCI, the IVUS-guided PCI group had significantly lower risks of MACE, stent thrombosis, cardiovascular deaths, target lesion revascularization, and target vessel revascularization. For complex PCI, IVUS reduces adverse events, importantly stent thrombosis and repeat revascularizations, compared with angiography alone guided PCI. A meta-analysis evaluates the PCI effects in patients with left main guided by IVUS or CAG. Compared to angiography alone-guided PCI, IVUS-guided PCI was associated with lower cardiovascular mortality and all-cause mortality as well as lower target lesion revascularization, MI, and stent thrombosis [33].

Collectively, these trials illustrate the promising role of IVUS in refining PCI for complex patients, including CHIP patients. The authors highlighted the ability of IVUS to optimize stent selection, sizing, and deployment, significantly reducing complications and enhancing procedural success rates (Table 1, Ref. [28, 29, 30, 31, 32]).

**Table 1.** **Clinical trials on IVUS guiding CHIP-PCI**.

First Author/Study	Sample	Study Design	Endpoints and Findings
Lee JM *et al*., 2023 [28] RENOVATE-COMPLEX PCI trial	1600 patients	Multicenter patients with complex coronary lesions were randomized at a 2:1 ratio to undergo either IVUS-guided PCI or CAG-guided PCI	The primary endpoint was a composite endpoint, which included cardiovascular death, target-vessel MI, or clinically driven TVR.
			IVUS-guided PCI demonstrated a 36% reduction in the incidence of TVF. There were no differences in procedural safety events between the two groups.
Hong SJ *et al*., 2020 [29] IVUS-XPL trial	1400 patients	Randomized trial, evaluate IVUS-guided PCI compared to CAG- PCI in patients for complex coronary lesions	IVUS-guided PCI significantly reduced MACE compared to CAG-PCI.
Kang DY *et al*., 2023 [30] The OCTIVUS trial	2000 patients	Randomized trial comparing the impact of IVUS and OCT on complex clinical outcomes	The primary endpoint and its constituent parts were less common in OCT images.
			Compared to OCT, IVUS more frequently satisfied the predefined imaging-guided stent.
Li X *et al*., 2024 [31] IVUS-ACS	3505 patients with ACS	Randomly assigned to IVUS-guided PCI or angiography-guided PCI	The primary endpoint occurred in 70 patients in the IVUS group and 128 patients in the CAG group, driven by reductions in target vessel MI or TVR. There were no significant differences in all-cause death or stent thrombosis between groups.
Kwon O *et al*., 2021 [32] CTO-PCI with DES	1077 patients with 1077 CTO lesions treated with DES	Evaluate the clinical benefits of performing post-stent IVUS in preventing adverse clinical events	Post-stent IVUS evaluation was associated with a lower risk of TLR. The final MSA was independently associated with TLR.

Note: ACS, acute coronary syndromes; CAG, coronary angiography; CTO, chronic total occlusion; DES, drug-eluting stent; IVUS, intravascular ultrasound; MACE, major adverse cardiovascular events; MI, myocardial infarction; MSA, minimum stent area; OCT, optical coherence tomography; PCI, percutaneous coronary intervention; TLR, target lesion revascularization; TVR, target vessel revascularization; TVF, target vessel failure; CHIP, complex higher risk-indicated patient.

### 6.2 Reduction in the Number of Adverse Events

The observational study by Choi *et al*. [34] marks a turning point in comprehending the pivotal role of IVUS in optimizing PCI for CHIP patients. IVUS significantly reduces complications such as stent malapposition and incomplete apposition by meticulously selecting stents and ensuring precise deployment. That study underscores how IVUS markedly enhances long-term outcomes by refining procedural success rates [34]. That study has limitations due to its nonrandomized design, potential selection biases, and inclusion of patients from a single center. Confounding factors such as IVUS use, treatment strategy, and vascular access could have influenced the results. However, this approach offers advantages such as long-term follow-up data collection and strict control. A comprehensive meta-analysis revealed a significant reduction in stent thrombosis and restenosis rates in CHIP patients who underwent IVUS-guided PCI compared to those who underwent angiography-guided procedures [35]. Precision stent optimization and identifying suboptimal stent deployment significantly mitigated the risk of adverse events post-procedure.

### 6.3 Enhanced Long-Term Outcomes

Observational data from a registry study by Lee *et al*. [28] highlighted superior long-term patency rates in CHIP patients who underwent IVUS-guided PCI compared to those guided solely by angiography. Reduced restenosis and stent thrombosis rates contributed to the improved durability of the interventions [36]. The precision of stent placement and optimization resulted in fewer instances of target lesion revascularization, indicating the sustained efficacy of IVUS guidance in clinical practice.

The accumulated evidence from studies, trials, meta-analyses, and real-world data unequivocally supports the efficacy of IVUS-guided PCI in CHIP patients [37]. The meticulous lesion assessment, precise stent sizing, and optimized deployment achieved through IVUS guidance consistently correlate with enhanced procedural success rates, reduced adverse events, and improved long-term outcomes. These findings underscore the pivotal role of IVUS technology in reshaping coronary interventions for CHIP patients, suggesting its integration into standard practice to optimize patient care and cardiovascular outcomes in this high-risk population.

## 7. Current Challenges and Strategies in Implementing IVUS-Guided PCI for CHIP Patients

The cost of IVUS catheters and additional procedural time can pose challenges in routine practice. Strategies such as cost-effectiveness analyses, workflow optimization, and selective use of IVUS in specific high-risk scenarios can mitigate financial burdens and procedural delays [38]. IVUS interpretation requires specialized skills, and operator proficiency is crucial. Ensuring comprehensive training programs, skill development workshops, and continuous education for interventional cardiologists can overcome this challenge, enhancing IVUS utilization. Limited data exist for specific subsets of CHIP patients, such as those with heavily calcified lesions or chronic total occlusions. Conducting dedicated studies targeting these subgroups and pooling data from real-world registries can expand the evidence base and guide tailored approaches. The routine integration of IVUS into CHIP cases might lead to resistance due to established practices and workflow challenges. Encouraging institutional protocols and clinical guidelines advocating IVUS use in high-risk scenarios and fostering a culture of evidence-based practice can facilitate its adoption [39].

Conducting comprehensive cost-benefit analyses to demonstrate the long-term advantages of IVUS-guided PCI for reducing complications, repeat interventions, and healthcare costs can justify its utilization in CHIP patients [40]. Continuous education, training programs, and simulation-based learning for interventional cardiologists can ensure proficiency in IVUS interpretation and encourage its widespread adoption. Developing algorithms or risk stratification tools to identify high-risk lesions or cases where IVUS provides maximum benefit can optimize its selective use, addressing cost and time constraints. Collaborating with professional societies to develop and endorse guidelines advocating IVUS use in specific high-risk scenarios within CHIP patients can standardize its integration into clinical practice. The advantages of IVUS-guided PCI in CHIP patients, including improved procedural accuracy, reduced complications, and enhanced patient outcomes, underscore its pivotal role in high-risk interventions. Acknowledging challenges related to cost, expertise, evidence gaps, and integration, strategic measures such as cost-effectiveness analyses, education, tailored use, and guideline advocacy can overcome these hurdles, paving the way for optimized utilization and improved care in this vulnerable patient population. The current clinical guidelines and recommendations established by prominent cardiology societies or associations concerning the utilization of IVUS in PCI for CHIP are described below.

## 8. Guideline Recommendations and the Integration of IVUS into Standard Practice

The recommended guidelines for IVUS-guided PCI are listed below. The American College of Cardiology/American Heart Association (ACC/AHA) guidelines acknowledge the role of IVUS in specific scenarios, recommending its use in selected complex lesions or when angiographic evaluation is inconclusive. However, specific recommendations for IVUS-guided PCI in CHIP patients are lacking. The European Society of Cardiology (ESC) guidelines emphasize the adjunctive role of IVUS in complex lesion assessment, particularly in patients with ambiguous angiographic findings or bifurcation lesions. While not explicitly focused on CHIP patients, the ESC guidelines advocate IVUS utilization in challenging cases. The Asian-Pacific Society of Cardiology (APSC) recommends the utility of IVUS for guiding stent implantation, especially under challenging lesions or in those with unclear angiographic images. While not explicitly tailored for CHIP patients, it supports IVUS use in challenging interventions. The Society for Cardiovascular Angiography and Interventions (SCAI) offers more specific guidance, highlighting the utility of IVUS in optimizing stent sizing, apposition, and lesion assessment in complex lesions. It acknowledges its potential to improve procedural outcomes but does not explicitly target CHIP patients [41].

IVUS integration significantly impacts clinical decision-making in CHIP patients. The detailed lesion assessment facilitates precise stent selection, sizing, and deployment, influencing decisions on optimal treatment strategies [39]. IVUS enables risk stratification by providing insights into lesion morphology, plaque characteristics, and vessel dimensions, aiding in tailoring interventions to individual patient needs, especially in the high-risk subset of CHIP patients [6]. Integrating IVUS into standard practice improves procedural outcomes by reducing complications, enhancing stent apposition, minimizing geographic misses, and aligning interventions more closely with the anatomical needs of complex lesions. IVUS-guided PCI decreases the number of adverse events, including lower rates of restenosis, stent thrombosis, and target lesion revascularization. Its integration positively impacts long-term clinical outcomes in CHIP patients.

## 9. Clinical Comparison between IVUS- or OCT-Guided PCI

OCT and IVUS have shown comparable outcomes in guiding PCI. A randomized trial [42] was conducted at 38 European centers. Patients with a complex bifurcation lesion identified by CAG were randomly assigned to OCT- or angiography-guided PCI. Whether OCT-guided PCI improves clinical outcomes compared to CAG guidance. At two years, a primary end-point event of the OCT-guided PCI group had a significant decrease compared to the CAG-guided PCI group. Among patients with coronary bifurcation lesions, OCT-guided PCI was associated with a lower incidence of MACE than CAG-guided PCI.

An intravascular imaging study [43] compared the effectiveness and safety of OCT- vs. IVUS-guided PCI for complex coronary artery lesions, which included unprotected left main disease, bifurcation disease, an aorto-ostial lesion, a chronic total occlusion, a severely calcified lesion, an in-stent restenotic lesion, a diffuse long lesion, or multivessel PCI. The primary endpoint was a composite of death. At a median follow-up of two years, the primary endpoint event in 2008 randomized patients had occurred in 47 patients in the OCT-guided group and in 56 patients in the IVUS-guided group (HR: 0.87; 95% CI: 0.59–1.29). The incidence of major procedural complications was lower in the OCT-guided group than in the IVUS-guided group. Among patients with complex coronary artery lesions, OCT-guided PCI showed a similar risk of a primary composite event of death from cardiac causes, target vessel-related myocardial infarction, or target vessel revascularization as compared with IVUS-guided PCI. Further, randomized clinical trials should compare clinical benefits in CHIP patients treated with PCI guided by IVUS- or OCT.

## 10. Conclusions

IVUS-guided PCI plays a transformative role in managing CHIP, enhancing precision, reducing complications, and influencing decision-making. Future research to refine IVUS strategies for specific CHIP subsets and comprehensive cost evaluations could further endorse its adoption and improve patient outcomes in complex coronary artery disease scenarios.
